# The efficacy of *Origanum majorana* nanocubosomal systems in ameliorating submandibular salivary gland alterations in streptozotocin-induced diabetic rats

**DOI:** 10.1080/10717544.2021.2018522

**Published:** 2021-12-29

**Authors:** Dina B. E. Farag, Carol Yousry, Abdulaziz Mohsen Al-Mahallawi, Hesham I. El-Askary, Meselhy R. Meselhy, Nermeen AbuBakr

**Affiliations:** a Department of Oral Biology, Faculty of Dentistry, Cairo University, Cairo, Egypt; b Department of Pharmaceutics and Industrial Pharmacy, Faculty of Pharmacy, Cairo University, Cairo, Egypt; c School of Life and Medical Sciences, University of Hertfordshire Hosted by Global Academic Foundation, New Administrative Capital, Cairo, Egypt; d Department of Pharmacognosy, Faculty of Pharmacy, Cairo University, Cairo, Egypt

**Keywords:** Diabetes mellitus, *Origanum majorana*, rosmarinic acid, HPLC standardization, cubosomal nano-vesicles, salivary glands

## Abstract

Diabetes mellitus is a challenging health problem. Salivary gland dysfunction is one of its complications. Current treatments possess numerous adverse effects. Therefore, herbal extracts have emerged as a promising approach for safe and effective treatment. However, they are required in large doses to achieve the desired effect. Accordingly, *Origanum majorana* extract (OE) was incorporated into nano-sized systems to enhance its biological effects at lower dosages. OE was standardized against rosmarinic acid (RA) and then loaded into nano-cubosomal (NC) systems via a 2^3^ full-factorial design. Two optimum nano-systems at different drug loads (2.08 or 1.04 mg-RA/mL) were selected and assessed *in vivo* to compare their effects in streptozotocin-induced diabetic rats against conventional OE (2.08 mg-RA/mL). Blood glucose was evaluated weekly. Submandibular salivary glands were processed for histopathological examination and nuclear factor-erythroid 2-related factor 2 (*Nrf2*), Kelch-like ECH-associated protein 1 (*Keap1*), and *p38-MAPK* gene expression analysis. NC systems were successfully prepared and optimized where the optimum systems showed nano-sized vesicles (210.4–368.3 nm) and high zeta potential values. *In vivo* results showed a significant lower blood glucose in all treated groups, with an exceptional reduction with NC formulations. Marked histopathological improvement was observed in all OE*-*treated groups, with OE-NC4 (2.08 mg-RA/mL) demonstrating the best features. This was supported by RT-PCR; where the OE-NC4 group recorded the highest mean value of *Nrf2* and the least mean values of *Keap1* and *p38-MAPK*, followed by OE-NC3 and OE groups. In conclusion, OE-loaded NC enhanced the anti-hyperglycemic effect of OE and ameliorated diabetic gland alterations compared to conventional OE. Thus, cubosomal nano-systems could be anticipated as potential carriers for the best outcome with OE.

## 1. Introduction

Diabetes mellitus (DM) is a multifactorial metabolic disorder. It is one of the most challenging unsolved health problems of the twenty-first century. Nowadays, more than 400 million people have DM. This number is anticipated to rise to 642 million by 2040 (Ogurtsova et al., 2017). DM is a cluster of metabolic disorders characterized by hyperglycemia, lipoprotein abnormalities, raised basal metabolic rate, disturbances in reactive oxygen species-scavenging enzymes, and elevated oxidative stress-induced damage (Kesavulu et al., 2000). Over time, poorly controlled DM may lead to increased macrovascular and microvascular complications and end-organ damage in various body systems. Several oral complications have been reported in previous literature, including periodontal disease, fungal infection, and salivary dysfunction (Al-Maskari et al., 2011).

The current treatment modalities for DM mainly depend on oral hypoglycemic drugs or insulin. In addition to being unaffordable, these drugs possess numerous adverse effects, such as hyponatremia, obstructive jaundice, nausea, headaches, vomiting, and weight gain (UK Prospective Diabetes Study (UKPDS) Group, 1998; American Diabetes Association, 2009). In both developed and developing countries, most people rely on herbal medicines for their primary health care, as they are often viewed as a balanced and moderate approach to treat chronic diseases. Recently, a number of standardized herbal products have been approved for treating DM and its associated complications (Campbell-Tofte et al., 2012).

*Origanum majorana* (in English: Oregano or sweet marjoram) is an aromatic herb of the mint family (Lamiaceae) that commonly grows in Mediterranean regions and is widely used in food, traditional medicine, and cosmetic industries.The herb shows carminative, antispasmodic, diaphoretic, and diuretic properties (Dogan et al., 2004). The leaves have been claimed to treat respiratory and gastrointestinal disorders, while its ethanolic extract demonstrates antioxidant, antimicrobial, and anti-inflammatory effects (Deans & Svoboda, 1990; Ezzeddine et al., 2001; Jun et al., 2001; Heo et al., 2002). The herb is additionally rich in volatile oil, flavonoids, and phenolic acids (Deans & Svoboda, 1990; Vagi et al., 2002). Previous *in vitro* and *in vivo* studies on the pharmacological effect of *O. majorana* extract (OE) demonstrated that it can be used in treating DM as it shows potent anti-hyperglycemic effects and normalizes different histopathological changes associated with uncontrolled blood glucose levels via its antioxidant, immunomodulatory, and anti-apoptotic functions (Lemhadri et al., 2004; Vujicic et al., 2015). In addition, it can modulate gene-expression that is related to glucose and lipid metabolism, resulting in lower lipid accumulation in the liver and improved dyslipidemia associated with type-2 DM (Soliman et al., 2016).

Subjecting herbal extracts to the nano-sizing procedure or incorporating them into nanostructures is one of the potential strategies to improve their efficacy via decreasing the necessary dosage, improving their bioavailability, stability, and solubility, as well as enhancing their cellular uptake and biodistribution for better targeting behavior (Gera et al., 2017). Concisely, nano-formulations of herbal drugs may result in enhanced pharmacological activities at lower doses when compared to free herbal drugs (Wani et al., 2015). Different types of lipid-based nano-systems have been developed to enhance the absorption and overcome the common drawbacks associated with conventional delivery systems of synthetic and herbal drugs. Among these, cubosomes may be considered as promising drug nano-carriers due to their great potential as an alternative delivery system to the conventional lipid vesicles, liposomes. Cubosomal nano-particles, especially the ones composed of binary systems of water and glyceryl monooleate (GMO), are the most investigated systems (Larsson, 1983). These systems could be regarded as hydrophilic surfactant systems that possess the capability to self-assemble as a bicontinuous cubic liquid crystalline phase (Bei et al., 2009). Cubosomal systems are distinguished by their viscous nature, large surface area, and high ability to incorporate hydrophilic, lipophilic, and amphiphilic drugs (Nylander et al., 1996). These liquid crystalline systems have been used for drug delivery due to their distinctive 3D nano-structure with hydrophilic and hydrophobic domains. In addition, their contents of the lipid phase are biocompatible, bioadhesive, and biodegradable (Al-Mahallawi et al., 2021). Previous studies revealed that the bioadhesive characteristics of GMO-based liquid crystalline assembly enhanced the cellular uptake and improved the pharmacological effect of resveratrol, a herbal chemoprotective agent (Abdelaziz et al., 2019). In addition, previous studies showed that oral administration of drug-loaded cubosomes resulted in enhanced drug absorption, prolonged half-life, higher bioavailability, and promoted pharmacological activity when compared to free drug suspension (Shi et al., 2017; Mohsen et al., 2021).

In view of these facts, and since there is little reported experimental data on the biological activities of *O. majorana* nanosystems. In addition, to the best of our knowledge, the use of nano-cubosomal (NC) systems to improve the biological activity of *O. majorana* has not been investigated yet. Hence, the goal of this study was to evaluate and compare the protective effects of the standardized OE as well as the optimum OE-loaded NC systems in reducing blood glucose levels and suppressing the submandibular salivary gland histopathological alterations in streptozotocin (STZ)-induced diabetic rats. The authors aimed to enhance the protective effect of OE and reduce its required dose via incorporation into nano-sized formulations, as this might help to provide a safe, economic, and efficient novel anti-diabetic medication. To achieve this goal, OE was prepared, standardized and loaded into NC vesicles. The formulated nano-systems were characterized and the independent formulation factors were optimized via a 2^3^-full factorial design to select the optimum systems with the smallest particle size (PS) and the highest physical stability at different drug loads.

## 2. Materials and methods

### 2.1. Chemicals

GMO, Tween 80 (T80), and Pluronic F127 (PF127) were purchased from Sigma-Aldrich Chemical Company (St. Louis, MO). Solvents used for high-performance liquid chromatography (HPLC) were purchased from Merck (Darmstadt, Germany), and rosmarinic acid (RA) standard was obtained from Apin Chemicals Limited (Compton, UK). Distilled water was further purified using solvent filtration kit (Agilent Technology, Waldbronn, Germany) and acidulated water was filtered through a 0.45 µm cellulose nitrate membrane filter (ALBET^®^, Hahnemühle, Dassel, Germany) and degassed in an ultrasonic bath before being used in HPLC analysis.

### 2.2. Plant material

A sample of the leaves of *O. majorana* was collected in July 2019 from the plant cultivated in the Experimental Station of Medicinal Plants, Faculty of Pharmacy, Cairo University, Giza, Egypt. Plant identity was kindly confirmed by Mrs. Trease Labib, Consultant of Plant Taxonomy at Ministry of Agriculture and the former director of El-Orman botanic garden, and a voucher specimen (no. 1.10.2019) was deposited at the herbarium of the Department of Pharmacognosy, Faculty of Pharmacy, Cairo University. In accordance with institutional guidelines, additional approvals were not required to conduct research on the plant.

### 2.3. Preparation of a standardized extract of *O. majorana* leaves

A sample (500 g) of the air-dried powdered leaves was extracted with 70% ethanol (4 × 5 L) by sonication for 20 min in the ultrasonic bath till exhaustion. After filtration, the extracts were combined and the solvent was evaporated under reduced pressure to give a dark green, viscous residue (113 g, 22.6% w/w). The residue was dissolved in 500 mL distilled water by sonication to give a standardized OE (1:1 ratio, each 1 mL contains 17.81 mg RA as determined by HPLC) and the extract was then kept at 4 °C till use.

#### 2.3.1. Preparation of a standardized OE for biological study

**Figure 1.**
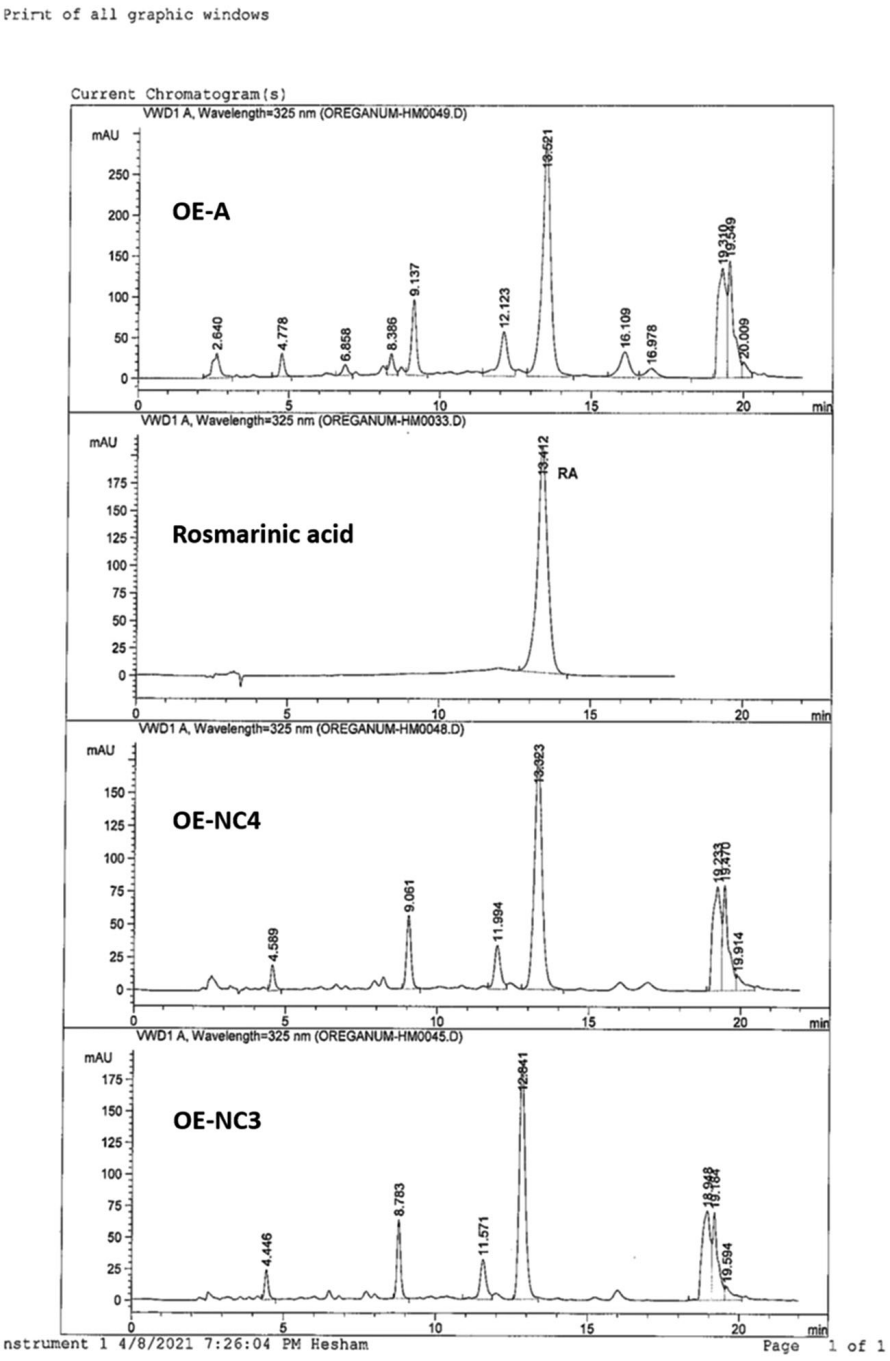
HPLC profiles of standardized OE and cubosomal dispersion formulas; OE-NC4 and OE-NC3.

A part of the standardized OE (17.5 mL) was diluted to 150 mL with water to give the OE; each 1 mL contains 2.08 mg RA (Figure 1).

#### 2.3.2. Preparation of cubosomal dispersion systems of OE for biological study (OE-NC)

An OE-loaded cubosomal dispersion was prepared where a 2^3^-full factorial design was adopted to evaluate and optimize the effect of the different independent formulation variables on the physicochemical properties of the formulated cubosomal systems. Three factors were studied at two levels each (Table 1); the OE load (*X*1; either low or high that is equivalent to 1.04 or 2.08 mg/mL of RA in the final dispersion, respectively), surface active agent (SAA) to lipid weight ratio (*X*2; 1:9 and 2:8) and finally, the SAA used (*X*3; either T80 or PF127). All eight possible combinations that are shown in Table 2 were performed in duplicates in a randomized manner to exclude the effect of time-related variables and satisfy the statistical requirements.

**Table 1.** Levels of the independent variables in the full factorial statistical design (2^3^) and the optimization criteria of the dependent variables.

Factors (independent variables)	Levels of variables	
	Low	High
*X*1: OE load (eq. to RA conc in final dispersion; mg/mL)	Low (1.04)	High (2.08)
*X*2: SAA:lipid weight ratio	1:9	2:8
*X*3: SAA type	Tween 80	Pluronic F127

Responses (dependent variables)	Constraints
*Y*1: PS	Minimize
*Y*2: PDI	–
*Y*3: ZP	Maximize in terms of absolute value

**Table 2.** Cubosomal dispersions corresponding to 2^3^-full factorial design with their resultant dependent variables.

Run number	OE load (equivalent to RA conc; mg/mL)	SAA:lipid ratio	SAA type	PS ± SD (nm)	PDI ± SD	ZP ± SD (mV)
OE-NC1	Low (1.04)	1:9	Tween 80	286.75 ± 38.96	0.387 ± 0.09	–37.60 ± 0.00
OE-NC2	High (2.08)	1:9	Tween 80	252.80 ± 2.83	0.227 ± 0.08	–43.05 ± 0.64
OE-NC3	Low (1.04)	2:8	Tween 80	210.40 ± 3.96	0.399 ± 0.04	–43.50 ± 3.68
OE-NC4	High (2.08)	2:8	Tween 80	368.30 ± 18.24	0.386 ± 0.01	–44.65 ± 3.23
OE-NC5	Low (1.04)	1:9	Pluronic F127	354.00 ± 1.98	0.677 ± 0.05	–32.05 ± 1.06
OE-NC6	High (2.08)	1:9	Pluronic F127	884.20 ± 306.6	0.139 ± 0.03	–28.40 ± 0.28
OE-NC7	Low (1.04)	2:8	Pluronic F127	157.00 ± 0.28	0.148 ± 0.03	–26.90 ± 0.28
OE-NC8	High (2.08)	2:8	Pluronic F127	539.80 ± 120.2	0.599 ± 0.27	–26.30 ± 0.14

Briefly, the NC dispersion of OE was prepared by emulsifying the oily phase within an aqueous phase (Esposito et al., 2003). The lipid phase; GMO, together with the SAA (composing 5% w/v of the total dispersion weight) were melted at 70 °C on a hot plate (WiseStir MSH-20D, Daihan Scientific, Seoul, South Korea). On the other hand, the specified volume of OE was dispersed in the aqueous phase and heated to the same temperature. Finally, the molten phase was dispersed in the pre-heated aqueous phase by stirring for 20 min, and the formulated NC dispersion was then sonicated for 10 min for further size reduction.

Analysis of variance (ANOVA) was applied to study the effect of the investigated formulation variables on the physicochemical properties of the formulated NC dispersion namely; Particle Size (PS; *Y*1), polydispersity index (PDI; *Y*2), and zeta potential (ZP; *Y*3) using Design-Expert^®^ Software (Version 7.0.0, Stat-Ease Inc., Minneapolis, MN). A *p* value <.05 was considered statistically significant.

##### 2.3.2.1. Particle size, polydispersity index, and zeta potential

The PS distribution of the formulated NC dispersions was determined by light scattering using Zetasizer Nano ZS (Malvern Instruments, Malvern, UK). Before measurement, NC dispersions were diluted 10 folds with double distilled water, at room temperature (30 °C), and placed in the chamber with a light scattering angle of 173°. In addition, the ZP values of the prepared systems were measured by a laser Doppler anemometer coupled with the same instrument. All measurements were repeated in duplicate (Ahmed et al., 2019).

##### 2.3.2.2. Selection of the optimal cubosomal nano-systems

After characterization of the formulated systems, the dependent variables were statistically optimized as per the constraints in Table 1 to select the optimal systems with the smallest PS, least PDI, and highest ZP for each drug load. The systems with the highest desirability factor were adopted for further investigations.

##### 2.3.2.3. Transmission electron microscopy (TEM)

The morphology of the optimum NC dispersions (OE-NC3 and OE-NC4) was observed using TEM (Jeol, 1200 EXII, Tokyo, Japan). A drop of the diluted dispersion was placed on a carbon-coated copper grid, followed by the addition of one drop of phosphotungstic acid (2%w/v aqueous solution). The sample was left to dry, and the excess reagent was removed using filter paper after 1 min. Finally, the grid was examined under a transmission electron microscope at 80 kV (Al-Mahallawi et al., 2019).

#### 2.3.3. HPLC quantification of RA

The amount of RA, the major phenolic acid in OE, and the optimum cubosomal nano-formulations (OE-NC3 and OE-NC4) were determined by HPLC.

For OE, 0.3 mL was transferred to a 50 mL measuring flask and the volume was made up to the mark with 80% methanol and sonicated for 5 min. For OE-NC3 and OE-NC4, 5 mL of the dispersion was transferred to a 25 mL and 50 mL measuring flask, respectively, and the volume was made up to the mark with 80% methanol and sonicated for 5 min. Each sample was separately filtered through a 0.45 μm membrane filter before HPLC analysis (Figure 1).

##### 2.3.3.1. Establishment of the calibration curve of RA

A standard stock solution of RA was prepared at a concentration of 150 µg/mL by transferring 7.5 mg of standard RA to a 50 mL measuring flask and the volume was made by methanol. Five serial concentration levels of RA in methanol were prepared in the range of 30–150 µg/mL. Twenty microliters of each concentration were injected in triplicates. The corresponding peak areas were recorded, and the calibration curve was constructed by plotting mean peak areas versus concentration. Linearity was assessed by linear regression method, calculated by the least square method; the correlation coefficient (*r*^2^) for the standard calibration curve was 0.997 and linearity of the peak area of RA was in the range of 10–150 µg/mL. The standard curve was used for the quantification of RA in the plant extract ‘OE’ and its cubosomal nano-formulations were used in biological testing; OE-NC3 and OE-NC4.

##### 2.3.3.2. HPLC apparatus

Agilent Technologies 1100 series, HPLC system (Agilent Technologies, Palo Alto, CA), equipped with a quaternary pump and degasser G1322A series 1200 was used. Agilent ChemStation software was used for data acquisition and processing.

##### 2.3.3.3. HPLC conditions

HPLC analysis was carried out on Lichrospher RP-C18 column (5 µm, 250 mm *L* × 4 mm ID, Merck, Darmstadt, Germany), preceded by a C18 guard column (5 µm, 10 mm *L* × 4 mm ID). The mobile phase composed of acetonitrile ‘solvent A’ and 0.3% H_3_PO_4_ in H_2_O ‘solvent B’ was used in the following gradient elution mode: 18% A/B to 25% A/B in 5 min, then to 27% A/B in 10 min and to 100% A in 2 min, continued for 4 min, and to 18% A/B in 3 min. Flow rate 1 mL/min, injection volume 20 µL, and UV detection was set at 325 nm.

### 2.4. *In vivo* studies

#### 2.4.1. Experimental animals

The experimental protocol was approved by the institutional animal care and use committee, Faculty of Science, Cairo University, Egypt (approval number: CU/III/F/31/20). Twenty adult male Albino rats with an average weight of 150–200 g and 3–4 months age were obtained from the animal house, Faculty of Medicine, Cairo University, Egypt. They were housed in an animal laboratory and were kept in wire mesh cages. The animals were kept in a controlled environment with a temperature of 23 ± 2 °C, a 12 h/12 h daylight cycle, and a humidity of 45–60%. Animals had access to a standard laboratory diet (crude protein 22%, crude fat 3%, crude fibers 3.9%, calcium 0.8%, phosphorus 0.4% and acid soluble ash 8%; Ibex International Co., Ltd, Giza, Egypt) and water *ad libitum.*

#### 2.4.2. Induction of DM

DM was induced in all the experimental animals by an intraperitoneal injection of STZ; 50 mg/kg body weight in 0.01 M citrate buffer at pH 4.5 (Zalewska et al., 2015). The diabetic state was confirmed in STZ-treated rats by measuring the blood glucose concentration after 72 hours. Rats with blood glucose levels greater than 200–250 mg/dL were used as diabetic rats. DM was allowed to be stabilized for seven days (AbuBakr et al., 2020).

#### 2.4.3. Experimental design

Twenty STZ-induced diabetic rats were randomly divided into four groups (*n* = 5/group) as follows:

- *Group I (DM):* received 1 mL of distilled water (once/day) by oral gavage for 4 weeks.
- *Group II (DM + OE):* received 1 mL of standardized OE (2.08 mg RA/mL) (once/day) by oral gavage (Hasanein & Mohammad Zaheri, 2014) for 4 weeks.
- *Group III (DM + OE-NC3):* received 1 mL of NC dispersion of OE (OE-NC3) (1.04 mg RA/mL) (once/day) by oral gavage for 4 weeks.
- *Group IV (DM + OE-NC4):* received 1mL of NC dispersion of OE (OE-NC4) (2.08 mg RA/mL) (once/day) by oral gavage for 4 weeks.

During the experimental period, the blood glucose levels of different animal groups were estimated every week. At the end of the experiment, all rats were euthanized by an intraperitoneal injection of ketamine (100 mg/kg) (Lairez et al., 2013). The submandibular salivary glands were quickly dissected. One side of the gland was fixed immediately in 10% neutral buffered formalin for histopathological analysis. The other side was stored at −70 °C after being frozen in liquid nitrogen and used for RNA extraction and gene expression analysis.

#### 2.4.4. Histopathological examination

Ascending grades of alcohol were used to dehydrate the formalin-fixed tissue samples (submandibular salivary glands), and xylene was used as a clearing agent. The samples were then embedded in paraffin wax. The prepared paraffin blocks were cut into slices of 5-µm thickness and stained with hematoxylin and eosin (H&E) (Suvarna et al., 2013). Sections were examined and photographed with Leica DM 1000 light microscopy and a camera using Leica Application Suite-LAS software in the Oral Biology Department, Faculty of Dentistry, Cairo University.

#### 2.4.5. Quantitative RT-PCR analysis

Total RNA was purified from homogenized tissue samples using RNeasy purification reagent (Qiagen, Valencia, CA). Five micrograms of total RNA was then reverse transcribed into cDNA using prim-script kit (Takara, Shiga, Japan). Gene-specific primer sequences used for amplification are listed in Table 3. Quantification of mRNA expression of target genes was done by using SYBR Green PCR Master Mix (Applied Biosystems, Foster City, CA). The thermocycling profile included 30 min of reverse transcription at 50 °C, 15 min of polymerase activation at 95 °C, and finally 50 cycles of; denaturation at 95 °C for 60 s, annealing at 60 °C for 60 s, and extension at 72 °C for 60 s (Salem et al., 2021). *Beta-actin* served as an internal control. Relative gene expression levels of nuclear factor-erythroid 2-related factor 2 (*Nrf2*), Kelch-like ECH-associated protein 1 (*Keap1*), and *p38-MAPK* were measured using the 2^–ΔΔCT^ method.

**Table 3.** Primer sequence of all studied genes.

Gene	Primer sequence from 5′ to 3′	Gene bank accession number
*Nrf2*	Forward: GAACTTGATGCCGTTCAGCC	XM_011419592.1
	Reverse: GTCTCCACAAGGAAAGTGAATC	
*Keap1*	Forward: GAGTCCAAGAAGTGCTCTAAG	XM_011433564.1
	Reverse: GTCAGGATCATAGCACTCAA	
*P38 MAPK*	Forward: AGTGGCTGACCCTTATGAC	NM_031020
	Reverse: CACAGTGAAGTGGGATGGA	
*β-actin*	Forward: TGACGAGGCCCAGAGCAAGA	XM_002751780.4
	Reverse: ATGGGCACAGTGTGGGTGAC	

#### 2.4.6. Statistical analysis

The data from both the blood glucose measurements and the RT-PCR results were expressed as mean ± standard deviation. Statistical differences between groups were assessed by one-way ANOVA test. A post hoc Tukey’s test was used for multiple pairwise comparisons. A *p* value <.05 was considered statistically significant. The statistical package (SPSS, version 15.0, Chicago, IL) for social sciences was used.

## 3. Results

### 3.1. Characterization of the formulated OE-loaded cubosomal dispersion

**Figure 2.**
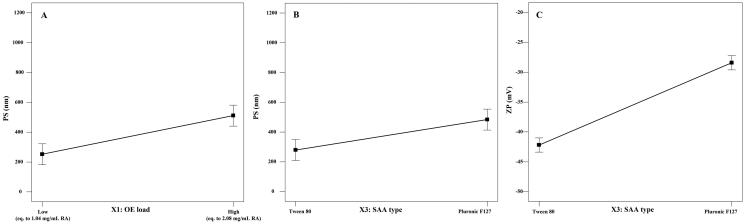
Line plot for the main effect of the OE load volume (A) and SAA type (B) on the PS of the formulated cubosomes, as well as the main effect of SAA type on the ZP values of the cubosomal dispersion (C).

PS and ZP measurements were performed to assure the nano-size and stability of the formulated NC dispersions. All the prepared systems showed nano-sized cubosomes with a mean PS distribution (by intensity) ranging from 157 to 884.20 nm and acceptable PDI values (less than 0.70) (Mudalige et al., 2019). ANOVA statistical analysis of the PS data suggested that increasing the OE content as well as the change in the SAA type (using PF127 instead of T80) showed a significant positive effect on the PS (*p* value <.05) as shown in Figure 2(A,B). In addition, significant interactions between the effects of the SAA type and the other investigated factors were observed. On the other hand, statistical analysis of the PDI values of the NC dispersions revealed that they were not significantly affected by any of the formulation variables (*p*>.05).

ZP values reflect the total charge on the surface of the formulated cubosomes to assess their stability (Radwan et al., 2020). Higher absolute ZP values indicate higher surface charge and consequently, greater inter-particle repulsion preventing their aggregation and confirming the physical stability of the preparations (Yousry et al., 2020). As shown in Table 2, all the formulated systems showed a negative surface charge with high ZP values ranging from −26.30 to −44.65 mV ensuring high physical stability of the formulated system and less tendency for aggregation (Han et al., 2008). ANOVA statistical analysis of the ZP values showed that the SAA type is the main independent factor that significantly affected the surface charge of the formulated NC, either alone or through an interaction with the other two factors (OE load and SAA:lipid ratio) at *p* values <.05 (Figure 2(C)).

### 3.2. Selection of the optimal cubosomal nano-systems

Optimizing the independent formulation variables was performed using Design Expert^®^ software based on the PS and ZP constraints, as shown in Table 1. It should be stated that PDI (Y4) was not taken into consideration, as it was not statistically affected by the different independent formulation variables (Al-Mahallawi et al., 2017). The software suggested OE-NC4 and OE-NC3 as the two optimum systems with desirability factors 0.861 and 0.855, respectively. The two optimum systems were formulated using T80 in a 2:8 SAA to lipid ratio but with different OE load; high (equivalent to 2.08 mg/mL RA) and low (equivalent to 1.04 mg/mL RA) for OE-NC4 and OE-NC3, respectively. Both systems showed nano-sized vesicles (210.4 and 368.3 nm) and high ZP (–43.5 and −44.65 mV) as shown in Table 2. The optimized systems were prepared and evaluated in triplicate to ensure the reproducibility of the results. These two formulations were used for subsequent evaluation.

### 3.3. Transmission electron microscopy

**Figure 3.**
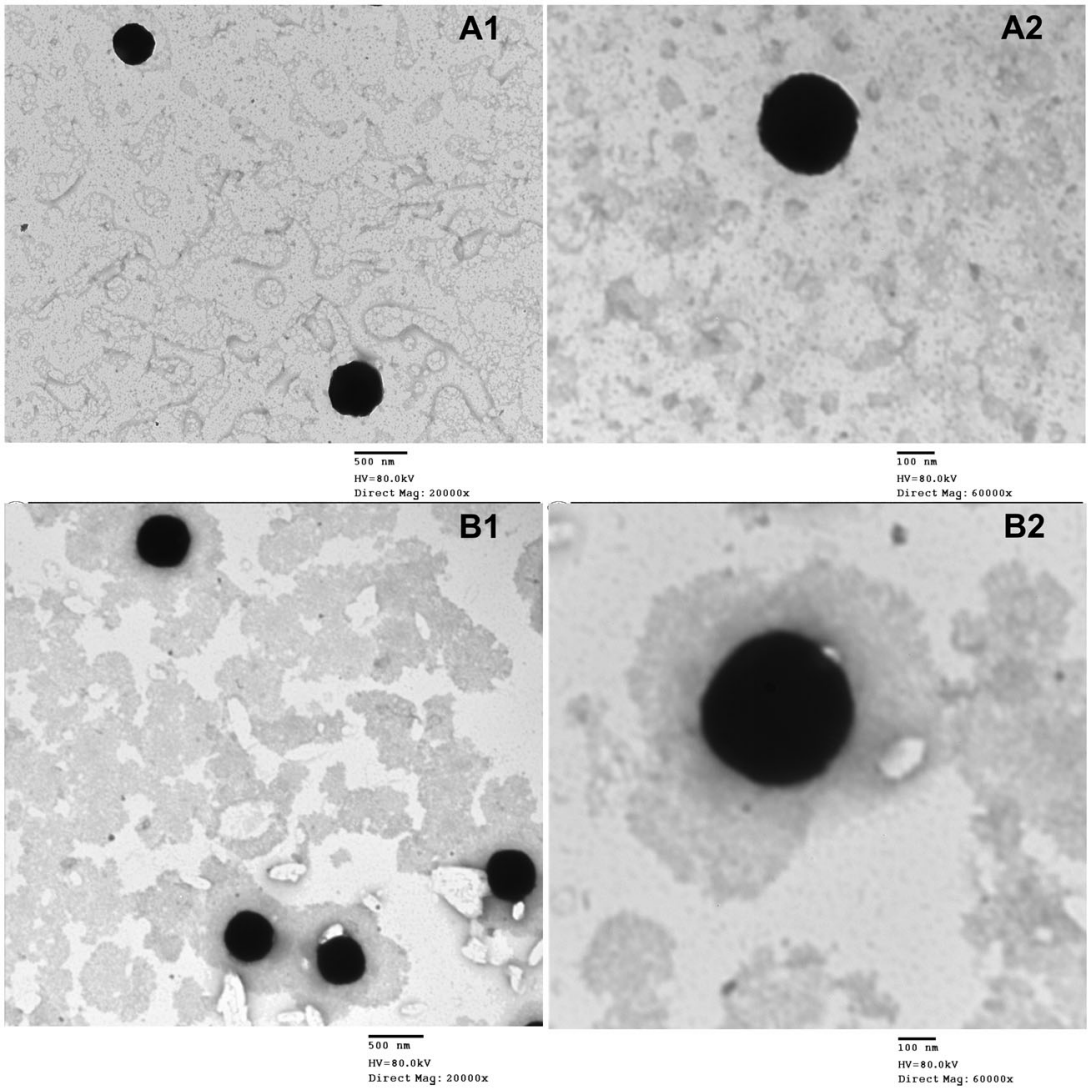
Transmission electron micrographs of (A) cubosomal dispersion OE-NC3 at different magnification powers (A1, A2) and (B) cubosomal dispersion OE-NC4 at different magnification powers (B1, B2).

The TEM images of the two optimum systems (OE-NC3 and OE-NC4) presented in Figure 3 showed well-dispersed, de-aggregated particles in the same nano-size range measured with the Malvern zetasizer (Malvern Instruments, Malvern, UK). The micrographs also showed the nearly cubic structure form of the formulated vesicles.

### 3.4. *In vivo* studies

#### 3.4.1. Effect on blood glucose level

The treatments with OE reduced the blood glucose levels in the diabetic rats. By comparing the blood glucose levels in all groups, the highest mean value was recorded in the diabetic untreated group (group I) and the lowest mean value was recorded in group IV (DM + OE-NC4 group). Statistical analysis using ANOVA revealed a significant difference between the four studied groups throughout the whole duration of the experiment (*p* value ˂.001). Multiple pairwise comparisons between the groups indicated that the OE-treated groups; DM + OE, DM + OE-NC3, and DM + OE-NC4 groups, respectively, exhibited a significant decrease in blood glucose levels compared to the untreated DM group at all time points (*p* value <.05). Additionally, the reduction in blood glucose levels observed in group IV (DM + OE-NC4) was significantly different from those observed in group II (DM + OE) and group III (DM + OE-NC3) at all time points (*p* value <.05). On the other hand, there was no significant difference between the blood glucose levels observed in group II (DM + OE) and group III (DM + OE-NC3) at weeks 1, 3, and 4 (*p* value >.05), whereas; the blood glucose levels observed in group III (DM + OE-NC3) were significantly lower when compared to group II (DM + OE) at week 2 (*p* value <.05) (Table 4).

**Table 4.** Mean ± SD of blood glucose level in all studied group.

Blood glucose level	Group I (DM)	Group II (DM + OE)	Group III (DM + OE-NC3)	Group IV (DM + OE-NC4)	*p* Value
Week 1	227 ± 1.5811^a^	221.2 ± 3.1145^b^	221.6 ± 1.8166^b^	207 ± 4.2426^c^	*p* ˂ .001**
Week 2	233.8 ± 4.3243^a^	219 ± 2.1213^b^	212.8 ± 2.2804^c^	201.8 ± 2.1679^d^	*p* ˂ .001**
Week 3	268.4 ± 7.4699^a^	142.2 ± 2.49^b^	142 ± 2.2361^b^	134 ± 2.5495^c^	*p* ˂ .001**
Week 4	307.6 ± 8.9889^a^	136.8 ± 1.9235^b^	131.8 ± 2.3875^b^	121.4 ± 3.9115^c^	*p* ˂ .001**

** Significant difference between all groups using ANOVA at *p* ˂ .001.

Means sharing different letters in the same row are statistically significant from each other using post hoc Tukey’s test.

#### 3.4.2. Histopathological examination of the submandibular salivary glands

**Figure 4.**
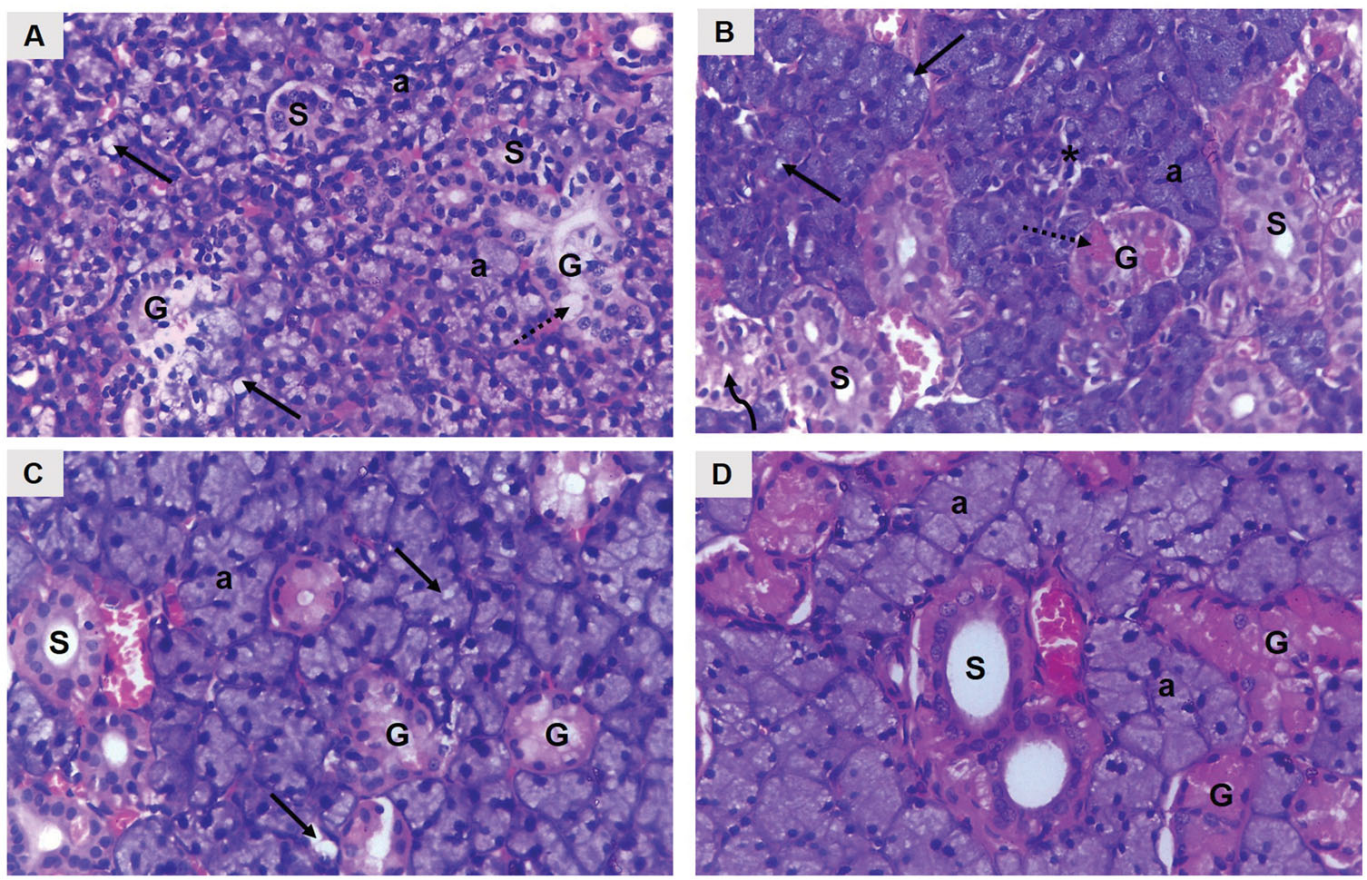
A photomicrograph of rat submandibular salivary gland showing (A) group I (DM): blurred acinar boundaries (a), massive vacuolization (arrows), degenerated granular convoluted tubule (G), ductal cell vacuolization (dotted arrow), striated duct with loss of basal cell boundary (S). (B) Group II (DM + OE): well defined acini (a), acinar vacuolization (arrows), absence of acinar configuration (asterisk), GCTs (G), clumped eosinophilic granules (dotted arrow), ductal vacuolization (curved arrow), striated ducts (S). (C) Group III (DM + OE-NC3): uniformly arranged acini (a), discrete vacuolization (arrows), GCTs (G), striated ducts (S). (D) Group IV (DM + OE-NC4): normal ductal parenchymal elements; acini (a), GCTs (G), striated ducts (S) (H&E, Orig. Mag. ×400).

**Figure 5.**
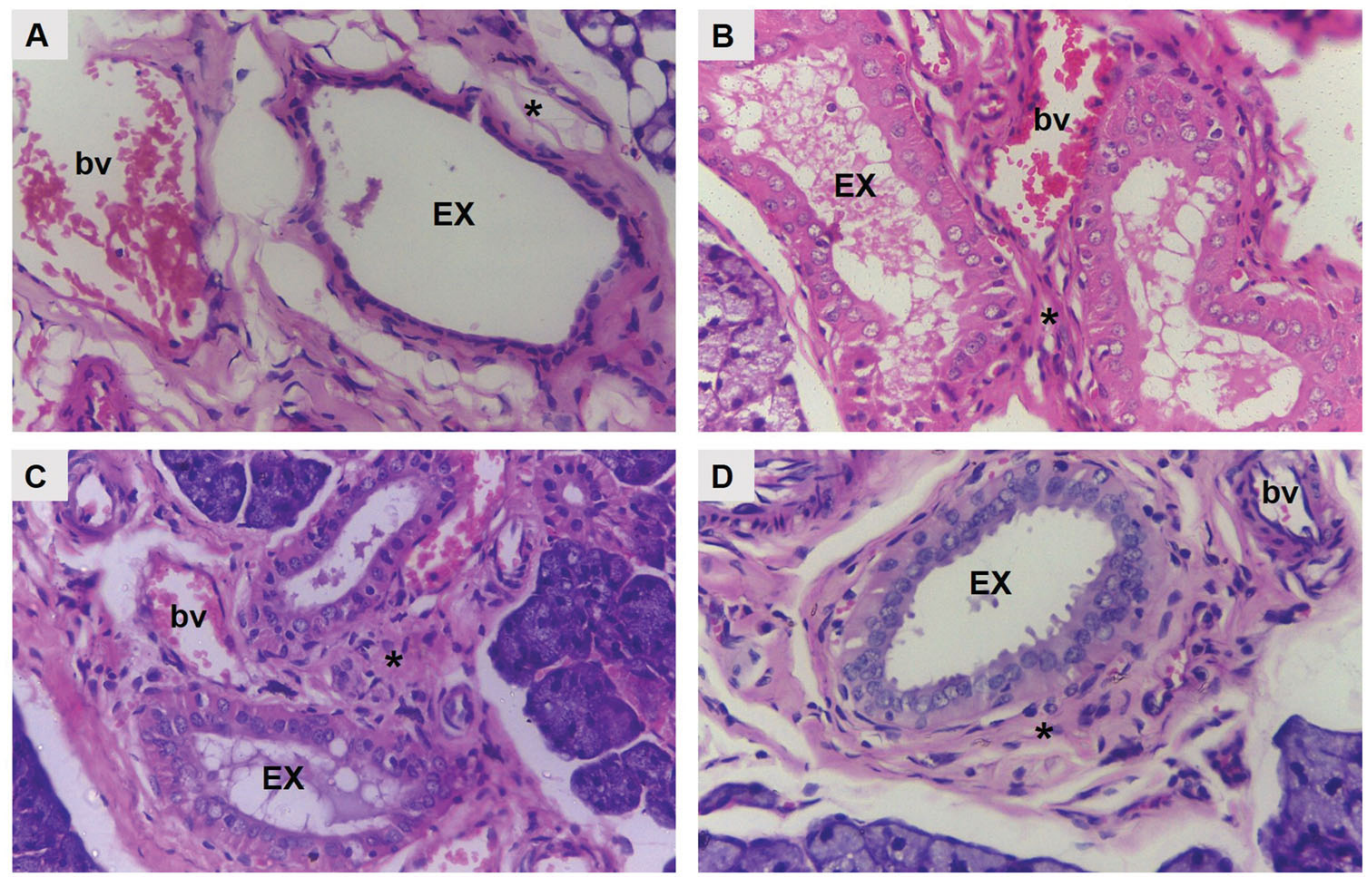
A photomicrograph of rat submandibular salivary gland showing excretory duct (EX), connective tissue stroma (asterisk) and blood vessels (bv) in (A) group I (DM), (B) group II (DM + OE), (C) group III (DM + OE-NC3), and (D) group IV (DM + OE-NC4) (H&E, Orig. Mag. ×400).

H&E-stained submandibular salivary gland sections of the diabetic untreated rats (group I) revealed severely distorted architecture. The glandular acini demonstrated loss of regular acinar configuration. Acinar blurred boundaries, loss of acinar cell septum, as well as massive cytoplasmic vacuolization were clearly demonstrated in most of the acini. Degenerative areas were evident in the granular convoluted tubules (GCTs) with obvious loss in cytoplasmic content as well as ductal cell vacuolization. The striated ducts showed indistinctly cellular basal boundaries with loss of basal striations (Figure 4(A)). Thinning and deformation of the epithelial lining of the excretory duct with loss of pseudo stratification were detected. The cell nuclei were flattened and apically displaced. Dissociated periductal connective tissue, as well as dilated congested ruptured blood vessel, was also observed (Figure 5(A)).

The treatments with OE ameliorated the histopathological alterations in the diabetic rats’ glands. The parenchymal elements of group II (DM + OE) appeared relatively well-organized compared to group I. Most of the acini showed well-defined cell boundaries. The acinar cell lining demonstrated basophilic cytoplasm with few cytoplasmic vacuolizations. However, some areas showed loss of regular acinar configuration. The GCTs presented relatively well-defined boundaries and well-arranged cells containing eosinophilic granules that were clumped in some areas with few ductal intracellular vacuolizations. Most of the striated ducts exhibited a normal cell lining with apparent basal striations (Figure 4(B)). The excretory ducts showed a well-defined boundary with a regular epithelial cell lining. Most of the nuclei appeared to be orderly arranged at different levels. However, stagnant secretion was evident throughout the ductal lumen. In addition, variable-sized blood vessels were observed in the connective tissue stroma surrounding the excretory duct. Moreover, the integrity of the endothelial lining was interrupted in some blood vessels (Figure 5(B)).

Regarding group III (DM + OE-NC3), the glandular acini were regular in shape and uniformly arranged. The lining cells showed basally located nuclei with basophilic cytoplasm. Discrete acinar vacuolizations were observed. The GCTs were lined with tall columnar cells having large rounded basally situated nuclei and discrete apical eosinophilic granules. Regular normal ductal configuration was observed in the striated ducts (Figure 4(C)). In addition, a normal epithelial lining, except for limited perinuclear vacuolization, was demonstrated in the excretory ducts. A few remnants of stagnant secretion were also observed in the ductal lumen. Periductal connective tissue stroma presented moderately dilated congested blood vessels with an intact endothelial lining (Figure 5(C)).

Finally, well-organized, normally architected parenchymal elements were observed in group IV (DM + OE-NC4). The acini demonstrated a normal configuration, lined by neatly arranged pyramidal cells, having granular basophilic cytoplasm and rounded basally situated nuclei. The acinar cells’ septum was clearly observed. The GCTs presented normal features with distinct ductal boundaries and orderly arranged lining cells, containing characteristic eosinophilic granules. The striated ducts were lined by columnar cells with rounded nuclei and intensely eosinophilic cytoplasm with basal striations (Figure 4(D)). A neatly arranged pseudostratified columnar epithelium was clearly observed lining the excretory ducts. The ducts were surrounded by a relatively well-organized fibrous connective tissue stroma containing regular normal blood vessels (Figure 5(D)).

#### 3.4.3. Quantitative RT-PCR analysis

The treatments with OE upregulated *Nrf2* and downregulated *Keap1* and *p38-MAPK* mRNA gene expression. Statistical analysis for mRNA gene expression of *Nrf2* showed a statistically significant difference among all groups using ANOVA (*p* value ˂.001), in which the highest mean value was observed in group IV (DM + OE-NC4) and the least value in the untreated DM group. Multiple pairwise comparisons between the groups indicated that the OE-treated groups; DM + OE, DM + OE-NC3, and DM + OE-NC4 groups, exhibited a significant increase in *Nrf2* mRNA gene expression compared to the untreated DM group (*p* value <.05). Constantly, the increase shown in the DM + OE-NC4 group was significant when compared to DM + OE as well to DM + OE-NC3 (*p* value <.05). However, no significant difference existed between DM + OE and DM + OE-NC3 groups (*p* value >.05) (Table 5).

**Table 5.** Mean ± SD of *Nrf2*, *Keap-1*, and *p38-MAPK* mRNA gene expression in all studied groups.

mRNA gene expression	Group I	Group II	Group III	Group IV	*p* Value
	DM	DM + OE	DM + OE-NC3	DM + OE-NC4	
*Nrf2*	1.0998 ± 0.1926^a^	2.2374 ± 0.2071^b^	2.5162 ± 0.4231^b^	3.6232 ± 0.5416^c^	*p* ˂ .001**
*Keap1*	2.318 ± 0.3773^a^	1.7262 ± 0.1746^b^	1.6832 ± 0.1198^b^	1.574 ± 0.2715^b^	*p* ˂ .05*
*p38 MAPK*	7.124 ± 0.2674^a^	4.212 ± 0.1763^b^	4.072 ± 0.1805^b^	2.65 ± 0.0583^c^	*p* ˂ .001**

*^,^**Significant difference between all groups using ANOVA (*p ˂ .05, **p ˂ .001). Means sharing different letters in the same row are statistically significant from each other using post hoc Tukey’s test.

Regarding *Keap1* mRNA gene expression level, the statistical analysis demonstrated a significant difference among all groups using ANOVA at *p* value <.05, with the highest mean value in the DM group and the least value in the DM + OE-NC4 group. Upon multiple pairwise comparisons, DM + OE, DM + OE-NC3, and DM + OE-NC4 groups showed a statistically significant decrease in *Keap1* mRNA as compared to the DM group (*p* value <.05). No significant difference was found between (DM + OE and DM + OE-NC3 groups), (DM + OE and DM + OE-NC4 groups), and between (DM + OE-NC3 and DM + OE-NC4 groups) (*p* value >.05) (Table 5).

As for *p38-MAPK* mRNA gene expression level, ANOVA analysis revealed a significant difference among all groups (*p* value ˂.001), where the highest mean value was shown in the DM group, while the DM + OE-NC4 group demonstrated the least value. Pairwise comparisons demonstrated that the decrease in *p38-MAPK* levels was significant among DM + OE, DM + OE-NC3, and DM + OE-NC4 groups in relation to the DM group (*p* value <.05). Additionally, *p38-MAPK* was significantly lower in the DM + OE-NC4 group than either DM + OE or DM + OE-NC3 (*p* value <.05). On the other hand, no significant difference was detected between DM + OE and DM + OE-NC3 groups (*p* value >.05) (Table 5).

## 4. Discussion

*O. majorana* is one of the medicinal plants that has been reported for its therapeutic potential in treating DM and suppressing its complications through different pathways (Campbell-Tofte et al., 2012; Tripathy et al., 2018). Nowadays, nano-sized systems have been widely used in drug delivery because they tend to improve the bioavailability of the administered drug, increase its residence time and allow better targeting behavior in the body which results in an enhanced pharmacological effect and/or concomitant reduction in the required dose (Mudshinge et al., 2011). The lipophilic nature of cubosomes has attracted special interest for their application as oral drug delivery systems. Being lipid-based vesicles, they show a significant role in drug absorption, which could be related to the ability of the lipids to stimulate pancreatic enzymes and bile secretion within the intestinal tract, where they enhance the formation of mixed micelles and improve the absorption rate of the loaded drug. Additionally, the inherent bioadhesive properties of cubosomes (especially with GMO in the lipid phase) allow close contact between the drug-loaded vesicles and the intestinal membrane, adding to their superior absorption characteristics (Karami & Hamidi, 2016; Yaghmur & Mu, 2021). Thus, the effectiveness of a standardized OE, either alone or after loading into cubosomal nano-systems at different concentrations, against the structural changes generated in diabetic salivary glands was assessed in this study.

To achieve this goal, a standardized OE was prepared, analyzed and accurately quantified to contain 17.81 mg RA per 1 mL of the extract. OE-loaded cubosomal nano-systems were then formulated at two different OE concentrations (equivalent to 1.04 and 2.08 mg/mL RA) via a 2^3^ full factorial design.

The PS data of the formulated NC systems revealed that the OE load exhibited a positive effect on the mean PS of the formulated NC dispersion, where increasing the OE load resulted in cubosomes with a larger PS. On one hand, this can be attributed to the load exerted over the cubosomes to pack higher amounts of the drug. On the other hand, it may be a result of the increased viscosity of the aqueous phase which may hinder the fine distribution of the ultrasonic waves and prevent the homogenous NC dispersion resulting in a larger PS (Pongpaibul & Whitworth, 1986).

In addition, cubosomes formulated using T80 as a surfactant showed a significantly smaller PS when compared to those formulated using PF127 (Figure 2(B)). A similar finding was observed by Musa et al. (2017). This could be explained in terms of the high molecular weight of PF127 compared to T80 that occupies a larger area and subsequently results in a higher NC size. Moreover, the small PS of the formulated T80-based cubosomes can be additionally explained by their high ZP values that generate a high repulsive force, preventing particle–particle aggregation, which may be encountered with PF127-based NC dispersion (Musa et al., 2017).

The negative surface charge of the NC dispersion is related mainly to the negative charge of the lipid (GMO) (Ebrahimi et al., 2015). As denoted by the statistical analysis of the ZP values, the use of T80 as a SAA resulted in NC dispersion with higher ZP values compared to those formulated using PF127 (Figure 2(C)), although both SAA types are nonionic surfactants that act mainly via steric stabilization. Similar results were formerly observed by Elmowafy et al. (2020). It was previously reported that the molecular weight, structure, and conformation of surfactants play a crucial role in determining the surface charge carried by the formulated vesicles, even with nonionic surfactants that act through steric stabilization (Kovacevic et al., 2011). Han et al. also demonstrated that although nonionic SAAs do not ionize into charged molecules, they can be adsorbed onto the surface charge of the water molecules at the particle/water interface, forming an electric double layer that is similar to the one formed with ionic SAAs (Han et al., 2008). In addition, the lower ZP values observed with PF127 can be attributed to its high hydrophilic lipophilic balance (HLB) value (22) (Seth & Katti, 2012) when compared to T80 (HLB value = 15) (Kassem et al., 2019). Ibrahim et al. (2015) observed that the ZP of the o/w emulsion decreases with the increase in the HLB values of the used nonionic SAA. They attributed their finding to the compression of the electrical double layer around the emulsion droplets, resulting in a reduced surface charge around the vesicles.

Upon optimizing the independent formulation variables at each OE-load, OE-NC4 and OE-NC3 were selected as the two optimum systems. Both systems were formulated using T80 in a 2:8 SAA to lipid ratio but at different OE concentrations as shown in Table 2. Finally, the free OE and the two optimum loaded nano-systems (OE-NC3 and OE-NC4) were examined *in vivo* in STZ-induced diabetic rats.

Histopathological examination of the submandibular salivary glands in the untreated diabetic rats (group I) showed severe glandular and cellular degeneration throughout the whole experiment. Similar cellular atrophy and cytoplasmic degeneration were previously reported in the salivary glands of diabetic rats (AbuBakr et al., 2020; Yasser & Shon, 2020; Salem et al., 2021). These results could be attributed to severe sustained hyperglycemic states in the diabetic model where high blood glucose levels were recorded.

It was proposed that hyperglycemia enhances the generation of free radicals (Çakatay & Kayali, 2006). Oxidative stress in turn induces damage to functional and structural macromolecules such as lipids, nucleic acids, and proteins and modulates the activity of antioxidant enzymes (Poljšak & Fink, 2014). The cell membrane is the first to be exposed to free radicals before the other cellular components undergo oxidative changes (Erel, 2005). This might explain the loss of cellular boundaries that was observed in both acinar and ductal cells in the present study. Moreover, the vascular endothelial cells are one of the main targets of hyperglycemic damage. Severe blood vessel injury demonstrated in the current study could be attributed to the inability of vascular endothelial cells to modulate intracellular glucose concentration with respect to blood glucose levels (Kaiser et al., 1993).

On the other hand, the diabetic rats treated with *O. majorana* preparations in all groups showed compact glandular architecture, and the degenerative changes observed in the untreated diabetic group were markedly suppressed. From these results, it could be assumed that the OE exerted a positive effect on the restoration of damaged cellular elements of salivary glands. These results are supported by a previous study by Soliman et al. (2016) who demonstrated that oregano extract enhanced hepatic parenchyma regeneration and restored the normal renal histological architecture in diabetic rats.

In accordance with previous studies (Soliman et al., 2016; Tripathy et al., 2018), the diabetic rats treated with *O. majorana* preparations in the current investigation revealed a significant decrease in their blood glucose levels as compared to the diabetic untreated group. The regulation of genes involved in glucose metabolism, such as adiponectin and glucose transporter-2 was proposed to be the mechanism through which OE reduces high blood glucose levels (Soliman et al., 2016).

Kelch-like ECH-associated protein 1-*Nrf2*-antioxidant response element signaling pathway is considered to be the most important antioxidant pathway (Lu et al., 2016). *Nrf2* is located in the cytosol where it is suppressed by its native inhibitor *Keap1*, which under unstressed conditions leads to *Nrf2* proteasomal degradation (Eggler et al., 2009). Upon exposure to oxidative stress, *Nrf2* is dissociated from *Keap-1* and is rapidly translocated into the nucleus to induce an antioxidant effect (Uruno et al., 2015). DM is associated with dysfunction in the *Nrf2–Keap1* signaling pathway, leading to inappropriate stress response (Soares et al., 2016). Given the implication of oxidative stress in the progression of DM as well as diabetic complications, *Keap1* and *Nrf2* mRNA gene expressions were investigated in the current study.

The RT-PCR results of the present study demonstrated that treatment of the diabetic rats with the *O. majorana* preparations was linked with a significant up-regulation of *Nrf2* gene expression as compared to the diabetic untreated group. This was concomitant with the down-regulation of *Keap1* gene expression. These observed results indicated that OE could be considered as a *Nrf2* activator agent. Since a wide range of diabetic complications were strongly correlated with a marked reduction in *Nrf2* gene expression, *Nrf2* activation is considered a promising strategy to attenuate these devastating complications (Zhong et al., 2013; Deliyanti et al., 2018). Histological results in the present study supported the hypothesis that *Nrf2* activation enhanced the proper cellular stress response, resulting in a cytoprotective effect.

A number of mechanisms through which *Nrf2* activating agents enhance *Nrf2* expression were suggested. It was demonstrated that down-regulation of *Keap1* could upregulate cytoplasmic *Nrf2* thereby, increasing the availability of nuclear translocation. Modification of *Keap1* cysteine residues, which in turn interrupts the *Nrf2–Keap1* complex and favors *Nrf2* release, was also reported. The blockage of proteasomal degradation of *Nrf2* is another proposed mechanism, whereas *Keap1* becomes saturated with *Nrf2* that cannot be degraded, and newly synthesized free *Nrf2* accumulates in the cell, resulting in free *Nrf2*, which is then translocated into the nucleus (Zhang, 2006; Bhakkiyalakshmi et al., 2015; Adelusi et al., 2020). Any of these previously reported mechanisms could be advocated in the present study. However, the exact mechanism that is responsible for *Nrf2* activation remains to be defined.

The *p38-MAPK* signaling pathway is an important member of the mitogen-activated protein kinases (MAPK) family (Raman et al., 2007). The activation of the *p38-MAPK* signaling pathway is responsible for the onset and development of diabetic complications (Thandavarayan et al., 2009) since it participates in apoptosis, immune regulation, and inflammatory reactions under oxidative stress (Zarubin & Jiahuai, 2005). Thus, in the herein study, mRNA gene expression of *p38-MAPK* was assessed.

Our results revealed that, compared with the diabetic untreated rats, the gene expression levels of *p38*-*MAPK* in the *O. majorana*-treated groups were significantly downregulated, indicating a reduction in the activity of this pathway. This in turn exerted a positive effect on the salivary gland tissue. In line with these findings, several studies have shown that tissue function can be restored in DM following the suppression of *p38*-*MAPK* activation. These effects were closely related to the significant decrease in inflammation, oxidative stress, and apoptosis following the inactivation of the *p38-MAPK* pathway (Zarubin & Jiahuai, 2005; Thandavarayan et al., 2009; Zuo et al., 2014).

The results of the current *in vivo* study clearly reflected the positive therapeutic potential of a standardized OE and its loaded cubosomal nano-formulations in a diabetic rat model. The biological activities of the OE observed herein might be related mainly to its active phenolic constituent, RA, as quantified by HPLC. In multiple studies, RA was found to have a wide range of bioactivities, including anti-inflammatory (Chen et al., 2018) and antioxidant effects (Ding et al., 2019). RA was also reported to induce glucose-lowering effect in STZ-induced diabetic rats by stimulating the surviving β-cells of the islets of Langerhans to secrete more insulin. It also reduced insulin resistance measures, indicating that the antioxidant capacity of RA helps to restore insulin sensitivity (Ramalingam et al., 2020).

However, the efficacy of the formulated OE-loaded NC systems outperformed the conventional formulation even at lower OE concentration. Interestingly, among the different NC systems prepared, the formula OE-NC4 (standardized to contain 2.08 mg RA/mL) displayed significantly lower blood glucose levels and better antioxidant and anti-inflammatory effects (marked with significantly higher *Nrf2* and lower *Keap1* and *p38-MAPK*) than the free OE at the same dose level (2.08 mg RA/mL), which showed comparable effects to that exerted by the formula OE-NC3 (standardized to contain half the OE load; 1.04 mg RA/mL). These findings support prior research, which revealed that nano-systems could improve drug absorption following oral administration by prolonging gastric residence time, improving mucosal bioadhesion, and increasing cellular uptake due to their small PS. They can also protect the loaded drug against gastric acidity, enzymatic and non-enzymatic degradation, as well as first-pass effect, which may result in a prolonged drug circulation half-life and improved pharmacological effect (Mudshinge et al., 2011; Kadam et al., 2012). All these factors may contribute to the superior performance observed with the OE-loaded NC formulation when compared to the conventional OE.

## 5. Conclusions

In this study, conventional OE and the two-optimum NC systems were tested *in vivo*. OE-loaded NC systems were successfully prepared with a small PS and a high ZP at different OE loads. Our results demonstrated that OE was effective in reducing the blood glucose levels and attenuating the histopathological and molecular alterations in the diabetic submandibular salivary glands through its anti-hyperglycemic, antioxidant, and anti-inflammatory effects. Additionally, it was shown that the biological activities of the extract were enhanced upon incorporation into NC systems, even at lower doses.

Since continuous research for developing novel, safe anti-diabetic drugs that could meet both health and economic needs of diabetic patients is mandatory, our findings provide a preliminary scientific base for the potential health benefits of using NC systems as an oral drug delivery system for OE in treating DM and ameliorating the associated structural changes. However, further experimental preclinical researches in the molecular efficacy, dosage, and safety related to these formulations are required for definitive authentication before application in human medical conditions.

## Data Availability

Not applicable.
